# Cases Occurring at the Clinic of the Jefferson Medical College

**Published:** 1848-01

**Authors:** James Patterson


### MEDICAL EXAMINER,

AND

### RECORD OF MEDICAL SCIENCE.

NEW SERIES.—No. XXXVII.—JANUARY, 1848.

### ORIGINAL COMMUNICATIONS.

### *Cases occurring at the Clinic of the Jefferson Medical College.*

Reported by JAMES PATTERSON, M. D.

MR. EDITOR:—Permit me from time to time to give publicity through the medium of your widely circulated journal, to some of the cases that present themselves at the surgical department of the Clinic of Jefferson Medical College, hoping that it may not be altogether uninteresting to some of your medical readers.

*Amputation of the Thigh, by Prof. Mütter.*

John M., æt. 14, residing in Pratt street below Spruce, a fine looking, intelligent youth, was admitted as an out-door patient to the surgical department on the 20th of Oct., with extensive disease of the left knee-joint, the result of an old white swelling.

The whole joint was enormously enlarged; the leg flexed at right angles with the thigh, and several large ulcers had formed in the vicinity, extending deep down into the joint and discharging a great quantity of fetid pus. According to his mother's account, the disease had existed for eleven years, and had been regarded as white swelling by several excellent physicians and surgeons, and treated as such; but it has only been two years since the ulceration commenced, and during that time his health has been rapidly failing. As there was hardly any probability of his ever obtaining the use of his limb, as his constitution was commencing to sink under the profuse discharges of purulent matter, and as he was still young, it was deemed expedient that the limb should be removed. On the 27th of October, the patient, having himself decided to submit to an operation, was carried into the amphitheatre and placed on the operating table, in presence of the class and several physicians. The patient was placed under the influence of sulphuric ether, by means of Dr. Roper's apparatus, and the double flap operation performed in the usual manner at the upper third of the thigh. The ether in this case was not wholly satisfactory, for during the latter part of the operation he screamed and struggled violently, as if suffering great pain,—although when questioned about it afterwards, he said he had no clear recollection of it, but it was all a confused dream.

The flaps having been brought together by means of sutures and adhesive straps, a pledget of lint, dipped in cold water, was then placed over the wound, and over this a piece of oil silk to prevent evaporation; then to confine the dressings and prevent retraction of the muscles, a broad roller bandage, thus making a light and clean dressing, calculated to bring about union by the first intention. The patient was placed in bed, and a tourniquet put around his limb to guard against hemorrhage, and perfect quiet enjoined.

Six weeks have elapsed since the operation, and the patient is at the present time perfectly recovered.

*Lithotomy—Simple Lateral Operation, by Prof. Pancoast.*

George Washington Groff, æt. 6, residing in West Philadelphia, was brought before the class on the 30th of October for the purpose of having a urinary calculus extracted from his bladder. The patient being placed upon the table, his hands and feet were tied together in the usual manner, and a staff having been introduced into the bladder, an oblique incision was made on the left side by means of a common scalpel, extending from the raphe to the middle of a line drawn from the anus to the tuberosity of the ischium, dividing the integuments, the perineal fascia, transverse perineal muscle and artery, membranous portion of the urethra, left lateral lobe of the prostate and neck of the bladder, the knife having been pushed down into the triangular space between the erector penis muscle and the accelerator urinæ, in order to reach the membranous portion of the urethra, cutting through some of the fibres of the levator ani muscle, and opening the urethra as near the base of the triangular ligament as possible, in order to avoid wounding the large artery of the bulb, which, coming off from the pudic, passes through the triangular ligament at a little distance below the symphysis pubis. The knife and staff having been withdrawn, a pair of forceps was thrust through the wound into the bladder, and the stone extracted, the whole operation not lasting more than three quarters of a minute, and performed with only three instruments, after the manner of Mr. Liston, whose great success as a lithotomist has been obtained by the use of the simplest apparatus, his incisions being commenced and completed with the same knife.

The patient was then placed in bed, laid on his left side, and a gum elastic catheter introduced into the bladder through the track of the wound, which, by facilitating the flow of urine, lessens the danger of infiltration and its dreadful consequences, and also presents an easy method of suppressing any venous or arterial hemorrhage.

Twelve hours after the operation a slight hemorrhage took place, which was easily restrained by the use of compresses dipped in cold water, and from that time to this (the twelfth day) the wound has been gradually uniting, and the urine has commenced to flow per urethram. The stone extracted was a mulberry calculus, very regular in its form, weighing one and a half drachms, and measuring three inches in its largest and two inches in its shortest diameter.

